# Seeing the Window, Finding the Spider: Applying Critical Race Theory to Medical Education to Make Up Where Biomedical Models and Social Determinants of Health Curricula Fall Short

**DOI:** 10.3389/fpubh.2021.653643

**Published:** 2021-07-09

**Authors:** Jennifer Tsai, Edwin Lindo, Khiara Bridges

**Affiliations:** ^1^Department of Emergency Medicine, Yale University School of Medicine, New Haven, CT, United States; ^2^Office of Healthcare Equity, University of Washington School of Medicine, Seattle, WA, United States; ^3^University of California, Berkeley School of Law, Berkeley, CA, United States

**Keywords:** critical race theory, health inequity and disparity, medical education, social determinants of health, biomedical model, health pedagogy, racial justice, medical critical race theory

## Abstract

A professional and moral medical education should equip trainees with the knowledge and skills necessary to effectively advance health equity. In this Perspective, we argue that critical theoretical frameworks should be taught to physicians so they can interrogate structural sources of racial inequities and achieve this goal. We begin by elucidating the shortcomings in the pedagogic approaches contemporary Biomedical and Social Determinants of Health (SDOH) curricula use in their discussion of health disparities. In particular, current medical pedagogy lacks self-reflexivity; encodes social identities like race and gender as essential risk factors; neglects to examine root causes of health inequity; and fails to teach learners how to challenge injustice. In contrast, we argue that Critical Race Theory (CRT) is a theoretical framework uniquely adept at addressing these concerns. It offers needed interdisciplinary perspectives that teach learners how to abolish biological racism; leverage historical contexts of oppression to inform interventions; center the scholarship of the marginalized; and understand the institutional mechanisms and ubiquity of racism. In sum, CRT does what biomedical and SDOH curricula cannot: rigorously teach physician trainees how to combat health inequity. In this essay, we demonstrate how the theoretical paradigms operationalized in discussions of health injustice affect the ability of learners to confront health inequity. We expound on CRT tenets, discuss their application to medical pedagogy, and provide an in-depth case study to ground our major argument that *theory matters*. We introduce MedCRT: a CRT-based framework for medical education, and advocate for its implementation into physician training.

## Introduction

As the healthcare system struggles to combat racial health injustices, it is important to interrogate how medical education may contribute by failing to address inequity on a pedagogical and rhetorical level (1, 2). Though a portion of US medical schools now include some health disparities teaching, few engage in critical examination of health inequity (3, 4). As defined by Kawachi, *health inequality* is the “generic term used to designate differences, variation, and disparities in the health achievements of individual groups,” whereas *health inequity* “refers to those inequalities in health that are deemed to be unfair or stemming from some form of injustice” (5).

This distinction between health *inequalities* (used here interchangeably with *health disparities*) and health *inequities* is important, and often missed. Many have expressed concern that current health disparities curricula—often referred to as “Social Determinants of Health” (SDOH) curricula—fail to engage with health inequity (6). These models merely name the existence of health differences and describe social determinants (such as access to food, educational attainment, income level) without relating them to power structures that marginalize different populations (6). This inability (or unwillingness) of SDOH to contextualize healthcare within relevant sociopolitical realities leaves trainees without Structural Competence—the proficiency to articulate or challenge root causes of unequal conditions (6–9). To ensure healthcare professionals are able to provide high-quality patient care and advance health justice, medical education needs a robust approach to health inequity that can scrutinize racial injustices pertaining to clinical practice, physician training, and scientific knowledge production (3, 10–12).

Critical Race Theory (CRT) is uniquely primed to help achieve this goal. CRT is an intellectual movement, body of scholarship, and analytical toolset historically developed to interrogate relationships between law and racial inequality (13). By training learners to identify and oppose fundamental sources of patient marginalization and engage in self-critique of health services research, CRT does what biomedical and health disparities curricula cannot: rigorously prepare physician trainees to combat health inequity. In this Perspective, we review current pitfalls of Biomedical and SDOH educational models, introduce MedCRT: A CRT-based framework for medical education, and advocate for its implementation in physician training.

## Background

How physicians are trained undoubtedly impacts the professionals they become and the systems they influence. Currently, medical training is dominated by knowledge produced by Western biomedicine, a field with limited diversity and inclusion (14–17). As such, the discipline has limited ability to disrupt social hierarchies (18) that perpetuate inequities, and has inadvertently reified problematic paradigms, including biological notions of race (3, 4, 8, 19).

Students and educators empowered as critical learners and scientist-scholars can create a more ethical healthcare system (4, 20). But US medical education notably lacks space—in terms of faculty positions, classroom hours, assessment considerations—dedicated to training students how political economy shapes medical knowledge and systems today (3, 11, 21). This undermines social science perspectives, effaces the muscularity of social powers in dictating health outcomes, and narrows critical scholarly introspection (22, 23). Biomedicine extolls the importance of peer review for strong scholarship, but fails to bring interdisciplinary experts to its own table (7).

Calls for anti-racist education have been made nationally (24, 25). However, these efforts have been mostly student-led or elective, and lack established support (8). Effective and critically anti-racist health justice education is not yet institutionalized in US medical education (3).

### What Is Your Theory? The Shortcomings of the Biomedical Model

The biomedical model (BM) characterizes bodies as machines, and disease as machine malfunction: pathology arises when biological components (hormones, tissues) are impaired (26–28). As Krieger states, this paradigm “divorce[s] external forces from the internal mechanisms: it focuses on the inner-workings of the machine…rather than interrogating factors that shape the contexts within which the machine acts (how was the machine built? Where does it thrive?)” (28). This focus on individual machinations relays that the source of disease—and disease disparities—is found within the body's borders, divorcing human health from socio-political realities (29). Because pathology is understood to arise from/in bodies, its presumed solutions do too. Proposed treatments engage only individual machines, and include pharmaceuticals, surgeries, and behavior changes (27, 29).

The BM is reductionist and essentialist. Because it reasons that risk factors are physiologic components of each patient's “machine” that confer higher probabilities of disease, ideas like race and sex are flattened as immutable characteristics that can be tabulated as machine parts. Rather than being appraised as complex political constructions rooted in cultural history, race becomes a series of genes; sex, a soup of hormones. This conceptualization deduces that health disparities arise from different and dysfunctional machine parts, which impedes nuanced comprehension of why people of different identities suffer unequal health outcomes (or what these identities even are). Learners of the BM discuss “poverty but not oppression, race but not racism, sex but not sexism, and homosexuality but not homophobia” (6).

Biomedicine has a set of assumptions and logic models—a theoretical paradigm—that guides how the discipline and its resulting scholarship conceive of concepts such as race, sex, disease disparities, and disability. This lens impacts the questions, methods, and conclusions produced. Yet, the field denies the existence of an overriding framework and presumes it is apolitical and value-neutral (3, 17, 30–33). It is a culture that cannot recognize its own culture (34). This hinders its ability to interrogate the window through which it observes and interprets the world (30).

All thinking and research is guided by theory, and critical formations within social science disciplines draw attention to this fact to interrogate the limits, format, and assembly of their “windows” and the views they provide (32, 35). This practice allows social scientists to analyze inequities that impact their approaches and explanations—their thinking (32). Lenses warp light. But biomedicine's insistence that it relies on pure empiricism—that it sees the world without an intervening theoretical frame—means it cannot evaluate the process, construction, and ensuing flaws of its understanding of bodies, disease, and racial difference (36–41). The field is left without the scholarly articulation and rhetorical defenses that help identify and combat racism embedded in hospitals and professional medical culture (32). This lack of reflexivity has ushered hidden curricula into the healthcare system (14–16). As result, medical trainees struggle to identify and disrupt injustice out in society *and* within their own professional homes. Given only the “Master's Tools” (42), they are left “on a road to nowhere” (6). Medical pedagogy needs critical perspectives that can help elucidate the social and scientific phenomena that allow injustice to perpetuate in its own house. It must learn to see the window, in order to critique and correct the lens through which contemporary biomedicine perceives racial inequities.

### Social Determinants of Health Theory: Where Is the Spider?

While many institutions have sought to address limitations of the BM with SDOH teaching, these emerging curricula are still ill-equipped to challenge health inequity (6). In these instances, *social determinants* are often presented as well-worn considerations that are in some ways natural and immutable. A number of conditions—poverty, race, diet, sex—are labeled as “risk factors” entangled on a “web” of contributing elements that increase the likelihood of a given pathology (27, 43–46). Importantly, however, the spider that weaves the web is absent in this metaphor (43). The material and historical conditions that create unequal distributions of power and resources—the conditions that spin the web of inequity such as racial supremacy, wealth concentration, neoliberal capitalism, and misogyny—are not included, considered, learned, or taught (43). The lack of an agent implies that these important determinants of health appear at nobody's behest. Thus, SDOH models imply that the disproportionate suffering of vulnerable populations originates from expected or natural differences instead of *inequities* engineered through unjust provisions set in place by empowered systems.

By pivoting discussions away from actors (spiders) that *create* inequity, SDOH curricula deliberate only biological, internal, and behavioral “causes” of disproportionate disease. Marginalized identities are pathologized: students learn that “urban” patients face more chronic disease due to poor diets and poverty. Learners are *not* taught unequal contexts of urban engineering, police surveillance, neighborhood segregation, and food deserts that limit well-being (47–49). This failure to include critical explanations on why, how, and by whom groups of people are historically and actively oppressed means SDOH curricula continually frame marginalized populations as deficient—not only financially, but also with regards to literacy, acumen, and ability (36, 37). Wealth and privilege are also social determinants of health—as are whiteness, citizenship, and proximity to political power. Yet, SDOH curricula bypass these considerations (and neglect to discuss who *benefits* from health inequity) to continually fixate on perceived deficiencies of “at-risk” populations. The repetition of the deficit-model constantly stigmatizes patients of color as poor, illiterate, needy, and unknowledgeable, while also implicitly supporting their surveillance, which can worsen existing health inequities (3, 4, 8, 19, 36, 37).

Without spiders, the visible agents in the web are patients, which locates them as the only active—and therefore, the only culpable—individuals in the disease pathway. Health disparities are thus framed as the outcome of poor individual choices or faulty genetics (4, 29, 38). This emphasizes targeted behavioral and biomedical interventions rather than considering structural solutions to structural obstacles. Consider, for example, how terms such as “non-compliant” (39, 40) ignore institutional inequities in insurance, transportation, and access that limit ability to adhere to prescribed treatments. This underscoring of individual culpability implicitly casts social justice efforts as philanthropic enterprises (needed to help people make better decisions or overcome genetic predisposition) rather than justified, reparative endeavors necessary to rectify historical wrongs (4). Like the BM, SDOH curricula lack theorization that explicitly associates socio-political economy with health inequity. Instead, students are taught to label populations as “vulnerable” without understanding what *makes* them vulnerable.

Lastly, because SDOH pedagogy does not incorporate teaching on actionable skills or solutions, students learn *about* health disparities but are not taught *how* to achieve justice (6). For example, implicit bias is framed as a cause of health disparities (13), but students are not asked to consider the *origins* of anti-black/pro-white biases, nor how to combat them. Prejudices are framed as subtle, innocuous preferences that are “unconscious,” which removes the purveyor's culpability and casts biases as normative and uncontrollable (41). Instead of an intervention, implicit bias tests—which associate racial discrimination with aversion to poisonous snakes—become “an alibi” that functions to permit further prejudice (50).

*Racism* is a leading cause of implicit bias: so how can students attack unconscious prejudice if they are not taught what racial hierarchy is or how it functions structurally? Without understanding how power operates in society, students cannot conceptualize how institutional and interpersonal prejudices disadvantage marginalized people regardless of conscious intent. White students, for example, will have difficulty comprehending racial inequity if they are unable to articulate or acknowledge their own privilege. This is also where traditional cultural competency models fail (51–54). Not only do they attempt to compartmentalize the needs of patient populations—often through troubling racial stereotypes—they “serve to further Other communities, because it (teaches) students to see difference without dissecting their own power” (17, 55). Learners receive information about human difference without being taught to challenge unjust distributions of power, which relegates SDOH pedagogy to a formality where competencies can be obtained without meaningful movement toward equity. Though medical schools may respond to the call for social justice with SDOH, lack of critical analyses on race and health renders these attempts ineffective (6).

## Critical Race Theory

We have described the ways US medical education fails to address health inequities. In their reticence or inability to engage in theoretical analysis of sociopolitical power, BM and SDOH curricula decontextualize human experience and erase patient perspectives (56, 57). Students are taught to fixate on individual choices and innate flaws—locating “responsibility” for poor health outcomes within those who inhabit them. Importantly, this may fail to engender empathy (or even discourage empathy) toward those facing structural violence (38). Lack of critical perspective also prevents “disciplinary self-critique” and fails to teach trainees how to act meaningfully against injustice (6, 12).

Current pedagogy on culture, bias, and diversity have been insufficient in engendering equity (54). New methods of building critical consciousness are necessary to bridge comprehension of inequities to care and praxis against them (58). A form of critical teaching and scholarship, Critical Race Theory (CRT) is able to address the deficiencies of current medical pedagogy by embracing tenets that help students achieve critical consciousness (59) of structural inequity (see Table 1).

**Table 1:** How critical race theory addresses deficiencies in existing medical curricula.

	**Deficiencies of the biomedical model**	**Strength of CRT that addresses pitfall**	
**Pathologize race**	Patient decontextualized, erasing individual patient perspectives	Utilize patient narratives with “Counter-Storytelling” and “Centering at the Margins”	**Theorize on and address health inequities: pathologize racism**
	Theorizes “Body as Machine,” casting race and sex as simple characteristics and Risk Factors inherent to individual physiology	Race seen as a dynamic, sociopolitical construct historically enforced to uphold power. Race is framed as a Risk Marker that indicates vulnerability to social inequity	
	Biomedicine is blind to its own theoretical paradigm; As a “culture that cannot recognize its own culture” it cannot critique its own window and theoretical perspective	Reflexivity allows CRT to consider internal power hierarchies that influence the construction of its scholarship and action; Sees and actively critiques the enmeshment of racial inequity in medical knowledge and practice paradigms	
	Proposed treatments and solutions only target individuals (Cannot propose solutions for broader social inequalities)	Proposed solutions target unique individual needs and address social and political inequity at large	
	**Deficiencies of SDOH curricula**		
**Theorizeon health inequalities: pathologize racial identities**	Web of Causation does not implicate causes of social inequity (Cannot see the spiders)	Emphasizes actors of power (spiders) that weave health inequity into society	
	View patients as only active agents; emphasize individual biological, internal, and behavioral interventions	Emphasize interventions on structures that create disproportionate burden of death and disease on vulnerable patients	
	Repeatedly uses Deficit Models to characterize vulnerable populations without discussing what makes them vulnerable (Ex. “Noncompliant patient”)	Acknowledges structural obstacles that create conditions that limit individual autonomy and ability to adhere to medical care	
	Do not teach on power and positionality; Students lack ability to think reflexively about the power of medical institutions and doctors in society	Requires learners to reflect extensively on power, positionality, and privilege	
	Frame healthcare inequity as aberrations/mistakes that can be fixed by optimizing current features	Frames the healthcare system as a fundamental source of inequity in America	
	Knowledge of healthcare disparities and inequalities is itself a measure of competence; Does not teach actionable skills to enact health justice	Equips learners with actionable skills and requires students to take active stances against health inequity	

Unlike the BM—which inaccurately presumes race is an essential component of the human machine—CRT asserts that race is not genetic but a power construct engineered to enforce racial hierarchy. In addition, CRT stresses that racism is so prevalent in society that it has become normalized to the point of invisibility (60–62). This recognition comes with comprehension that racism has shaped governing systems of the United States and is embedded into every institution of power (63). Thus, *normal* institutional operations—including that of medicine, healthcare, and scientific research—produce and perpetuate racial hierarchies and injustices *by design* (62). CRT demonstrates that racial categorization incites racism, which directs power and privilege toward some and away from others, justifies unfair outcomes, and reconciles how professed commitments to equality co-exist with the undeniable fact of injustice (13). This acknowledgment helps visualize the sociopolitical powers that influence medicine, and in juxtaposition to existing biomedical and SDOH models, grants learners and educators skills of self-critique required to “see the window” and dissect the racial inequities embedded in their own organizations (64). At its core, CRT seeks to identify and rectify systemic practices that generate racial injustice (63, 65). Importantly, CRT is not only interested in scholarship for scholarship's sake; It is committed to action that advances social equity (66, 67).

A medical CRT (MedCRT) framework requires scholars and learners to: “(1) Analyze race and racism as fundamental social structures within science, medicine, and society, (2) Challenge scientific theories of race that obscure the institutional mechanisms that generate racial health inequity, and (3) Produce analyses that mobilize and support antiracist praxis” (68) (see Table 2). MedCRT's iterative methodology continually questions complex power dynamics and “place[s] medicine in a social, cultural, and historical context” to develop nuanced comprehension of race, injustice, and health (51). While biomedical and SDOH models characterize race as an intrinsic individual risk *factor*, CRT asserts that racial identity should instead be understood as a risk *marker* that inscribes vulnerability to structural racial inequities in education, environmental safety, criminal justice, housing segregation, and social investment (45, 46). This transforms the question of differing racial epidemiology from “How do, and which intrinsic biological racial differences cause health disparities?” to “How do, and which racial injustices cause health inequities?” This allows robust avenues for learners to identify and intervene where health injustices originate. Further, in interrogating how and why atavistic beliefs of racial biology persist, (What is considered legitimate scientific knowledge? Who has the authority to create it? What agendas are implicitly supported by theories of intrinsic racial biology?) CRT not only allows for examination of biomedicine's theoretical window—it also abets understanding of how injustice has warped the lens (3).

**Table 2:** Critical race theory adapted to medical education (MedCRT) (13).

	**Critical race theory (CRT)**	**Application to medical education (MedCRT)**
**Race as a social construction**	**Scientific consensus demonstrates that race is not a genetic variable** (69, 70). Genetic differences among humans do not function to divide them into discrete biologic categories. Instead, genetic difference is a spectrum, making the demarcation of racial boundaries in the human population arbitrary. Phenotypic differences often used to construct racial categories—like skin color, hair, texture, eye shape, and lip size—do not reflect meaningful genetic inferences. Indeed, racial labels were originally mobilized to enforce racial hierarchies for colonialism (69–72). Foundational to CRT is the understanding that while the notion of biologic racial essentialism is erroneous, socially-constructed racial labels have powerful material consequences. Racial inequity determines proximity to illness and health, ultimately influencing who lives vs. who dies (73).	Despite scientific consensus that racial categories cannot be used to make meaningful genetic inferences, medicine continues to pathologize race as an immutable biologic variable (70, 71, 74). By using race as a scientific taxonomical tool, medicine reifies essentialist notions that frame bodies of color as “abnormal” variants of White bodies, which are normatively ascribed as “standard” (70, 71, 74). **This major CRT tenet requires that physicians reject education, research, and practice modalities that frame race as a genetic variable or fail to challenge racial essentialism**. Race corrections—such as those included in kidney and pulmonary function measurements—should be critiqued and used cautiously. Clinical training should explicitly instruct learners to understand race as a sociopolitical construct and inform them of the harms created when race is portrayed as a biologic trait that can used as a proxy for genomic information.
**Critique of colorblindness**	**CRT rejects the liberal embrace of colorblindness as the path to racial equity**. The critique lies in the premise that colorblindness narrowly conceptualizes racism as race-consciousness. Racism is not “thinking about race;” racism is “thinking about race *in the service of white supremacy*.” Colorblindness proposes that racial justice is achieved when everyone is treated similarly. CRT understands that this is an active injustice and seeks to treat marginalized people differently to guarantee equity.	Within the context of an unequal healthcare system that boasts rampant racial inequity, treating all patients equally merely maintains the status quo. The allocation of care and resources must be proportionate to injustice experienced. In addition, while race-based medicine that relies on biologic determinism should be critiqued, research utilizing racial labels to document racialized epidemiological inequities is important. **Medicine should not be color-blind, but race conscious in thoughtful and nuanced ways**.
**Intersectionality/anti-essentialism**	In 1989, Professor Kimberlé Crenshaw articulated the concept of Intersectionality to explain that an **individual's multiple positions** (regarding socioeconomic status, gender, citizenship, etc.) **must be interrogated to comprehend unique manifestations of racial subordination**. This also strengthens the claim of anti-essentialism; that there is no single experience for a given identity; there no common position to all “women,” “black people,” “trans people,” etc. (75). This supports the act of “Centering at the Margins,” in order to ensure that the unique needs of individuals who face intersecting oppressions from multiple axes of identity are addressed (76, 77).	Intersectionality appreciates the significance of layered identities in medical care. **Interrogation of different marginalizing forces that act simultaneously upon patients allows greater precision**. For example, impoverished Black women confront barriers that wealthy Black men do not face. Anti-essentialism also rejects problematic assumptions that people of the same identity have the same attitudes, experiences, and biology. It requires that physicians access humility and do not assume that they know the needs of each patient. To act otherwise is reductionist, intellectually unsound, and robs patients of the right to be seen and humanized as complex individuals.
**Ubiquity of racism**	**CRT identifies that racism is embedded into the everyday institutions that rule American society**. This integration of racial injustice into powerful governing bodies means that they will continue to reproduce and engender inequity through their normal function (13). Thus, sociopolitical apparatuses of power do not prioritize the interests of racially marginalized communities, and will only seek to accommodate inequities during conditions of Interest Convergence, wherein dominant groups are incentivized to act for their own benefit (78). This tenet recognizes the reality that goodwill alone fails to advance racial equity. In parallel, CRT vigorously critiques ahistoricism and seeks to understand how racism influences social, economic, and historical contexts that produce unequal realities (79). It recognizes that narratives of marginalized people have been excluded from history and formal scholarship, and advocates for “counter-storytelling” in order to center these epistemologies.	**Medicine is not immune to the primacy of racism**. Students must acknowledge that racial injustice is woven into the fabric of society to comprehend the depth of health inequity. Critical Consciousness is required for learners to discover and rectify personal and structural racial biases. Because unequal health is not an aberration, but an engineered result of our systems, progress cannot be achieved through passive scholarship. Explicit action is required to combat health injustice, because healthcare has omitted the needs of marginalized patients. Alongside the principle of Interest Convergence, active stakeholder involvement is necessary since medical institutions will not expand racial equity without explicit incentives. Though action may not further career promotion (since dominant powers do not have pre-existing motivation to prioritize equity), health justice must be sought by healthcare professionals—especially White colleagues who have greater privilege—as a moral obligation.

This sharpening of self-critique is not only important for the training of scientists and scholars who must examine the questions, methodologies, and interpretations of health inequality research to create new and better knowledge (12). It also aids in the Structural Competence and compassionate caregiving of clinicians. Both academic and clinical medicine are strengthened by the ability to understand one's position of power, as “critical consciousness” is theorized to be an important component of a trainee's ability to address health inequities (6, 80).

While traditional medical education may erase reflexivity by endorsing “the belief that (a healthcare provider's) class, race, ethnicity, gender, and sexual orientation are irrelevant to their medical practice” (17), CRT emphatically names its importance to ensure learners contextualize their care and improve on their ability to humanize patients (14, 81). For example, providers seeking to understand racial HIV inequities noted a “CRT lens proved especially useful in articulating the deep, complex, and systemic structural underpinnings of psychosocial barriers” in their patients, which allowed them to offer better, more compassionate medical care (82). This may ultimately improve patient outcomes, which represents an important opportunity for future research.

The guiding theory of illness (what causes and distributes disease) dictates the measures, methodologies, and justifications trainees and educators have to not only research and explain phenomena, but to articulate causative factors and thus imagine solutions (26–28, 38). Thus, theory matters. The window matters (see Box 1 for a case study). Overall, because CRT develops in learners a better understanding of structures of oppression, self-critique that can cultivate greater consciousness for change (105), and action-oriented praxis, it is a pedagogical intervention uniquely equipped to bolster health justice training and advancement (82). As a critical framework that offers necessary perspectives on race, racism, and health inequity for physicians, we propose that MedCRT should be employed to reform medical education.

Box 1 **Theory matters: racial asthma inequities as a case study.**

The guiding theory of illness dictates how students understand, explain, and challenge health inequities. Thus, theory matters. To reiterate our arguments in this paper and illustrate the importance of CRT-based medical curricula, we utilize the case example of childhood asthma, for which there are known contemporary health inequities. We show how Biomedical, SDOH, and CRT models of medical education identify different causal justifications for this racial difference in disease morbidity and mortality, and how these operationalized theories impact the corollary solutions each pedagogic framework proposes to address the problem. Concepts are summarized below.

For Black children, the mortality rate for asthma hangs six times higher than it does for their white counterparts (83). As discussed, the biomedical model portrays race as an essential genetic characteristic, and intuits that black race is an internal risk factor that predisposes black youth to this disease. As such, purveyors of biomedical theory may teach medical students about differential racial genetics that act as biologic predictors to asthma (84). Research on genetic mutations—such as those impacting SPINK5, DPP-1, and GRPA genes—are offered as the root of health disparities (85, 86). Genes that cause racialized responses to treatment options are also touted as rationale (87). The underlying notion is that if physician-scientists are able to locate genetic racial differences, targeting them through pharmacology and gene therapies will serve as a potent method to minimize racial disparities. This theory is pervasive and financially well-supported. The 2013 NIH Biennial Report (88) details million-dollar investment in the development of the “African Power Chip—” a genome-sequencing endeavor meant to “discover genes associated with asthma in African ancestry populations.”

SDOH curricula may go a step further in their discussion of asthma health inequalities by outlining a web of causation that connects risk factors like race, gender, and housing to unequal disease outcomes. It may teach students, for example, that people of color have higher exposure to mold, low-quality housing, or cockroaches that increase likelihood of asthma (89, 90). Data demonstrating that people of color have higher rates of smoking (89) may also be posited as a cause of disproportionate illness. Solutions, therefore, focus on behavioral changes like smoking cessation measures, patient outreach on hygiene education, or instruction to purchase hypoallergenic materials to minimize exposure to triggers. This ignores data that demonstrates how communities of color are targeted with significantly higher rates of tobacco advertisements as a predatory business strategy (91). SDOH shows students the web of health inequality, but not the spider that weaves racial inequity.

In fact, systemic racism manifests in myriad ways to cause racial health inequities in asthma. CRT helps us see how. This in turn helps inform solutions that are fundamental to promoting health justice. First, CRT asserts that race is a sociopolitical construct, which weakens the biomedical theory that differences in asthma prevalence can be explained by genetic racial differences in cell signaling and lung physiology. This also undermines the idea that genomic pursuits like the African Power Chip represent sustainable remedies to health inequities. CRT's position on the ubiquity of racism, the rejection of colorblindness, and the importance of intersectionality also combine to provide important insights that inform the causation of and interventions for asthma health inequities.

Given CRT's origins in law, it is appropriate to begin with the historical enmeshment of racial inequity in the criminal justice system. Today, the United States imprisons a larger percentage of its Black population that South Africa did *during apartheid* (92). Despite similar rates of drug use, Black men are 12 times more likely to be arrested for drug offenses than white counterparts (93). Being locked up is bad for your lungs. People with a history of incarceration are twice as likely to have asthma than the non-incarcerated American population (94).

America's lack of a socialized healthcare system ties medical access to financial security. While the black- white income gap itself is large, it is perhaps more important to consider the generational *wealth* gap, which is startling at 91,000 dollars for whites, 6,500 dollars for blacks. This is a 14-fold difference, and this gap is widening (95). Given economic analysis demonstrating that 50% of the median homeowner's wealth comes from the value of their property, it is important to understand how Black American families were historically denied home ownership. In the 1930's, the Federal Housing Association financed 60% of all American homes, yet <2% of these loans were awarded to people of color (96). Black neighborhoods were routinely “red-lined” and coded for mortgage default, stranding them in poorly-resourced and underdeveloped geographic locations (97). This discrimination is foundational to government-sponsored racial segregation, which, even when controlled for income, is tied to not only asthma, but heart disease, cancer, and lower life expectancy overall (98). In the South Bronx, a child is 14.2 times as likely to be hospitalized for asthma-related complications than a child in wealthier neighborhood <2 miles away (99). Importantly, the racial housing inequity is tied not only to issues of socioeconomic status, but environmental exposure.

Urban planning in America has time and again chosen to destroy places where people of color live, breathe, play, and pray. Throughout American history, black neighborhoods have been decimated to make way for highway construction, or else chosen as sites near which toxic waste landfills are placed (98, 100). Indeed, Black and Hispanic populations have higher exposure to 13 out of 14 main environmental pollutants (101) and are twice as likely to live near sources of industrial pollution in residential areas known as “sacrifice zones” (102).

CRT shows how the pervasiveness of racial injustice in American incarceration, urban planning, resource allocation, and environmental damage represent disproportionate, constant, and serious insults that are definitively linked to higher rates of lung disease in people of color. Its relevant intersections with poverty, imprisonment, and gender give methods to theorize thoughtfully on how to attend to specific populations jeopardized by multiple identities. Lastly, alongside epidemiologic scholarship on Weathering and Embodiment (103, 104)—concepts that tie how racial discrimination and social inequality translate to biologic dysfunction—the importance of rejecting to colorblindness as a path to equity is highlighted. It is necessary to pay attention to race insofar as it lets us see how racism is a major driver of health inequity. We need a critical theory of race—CRT—to locate the spider.

**Table:** 

**Model**	**Explanation**	**Solution**	**Interpretation**
Biomedical (BM)	Immunological dysfunction genetic racial difference biomarkers	Pharmaceuticals Genetic technologies, Racialized treatment algorithms	Race as an internal risk factor; racial physiology as culprit
		Ex. African Power Chip	
Social determinants of health (SDOH)	Web of causation	House cleanliness Patient outreach “Healthy Habits” Hypoallergenic Materials Mindfulness	
	Race, housing, air pollution, poor access to healthcare		
	Implicit bias		
Medical critical race theory (MedCRT)	Neighborhood segregation, federal housing association (FHA) policies	Political advocacy environmental regulations housing reform	Race as an external risk marker; racism as culprit
	Environmental racism and “Sacrifice Zones” built environment; highway distribution two-tiered medical system		
	Weathering, embodiment		

## Discussion

Critical Race Theory (CRT) emerged in the 1970s to challenge the shortcomings of the law by mobilizing an unrealized imagination: “What would the legal landscape look like today if non-white people were at the table when our society and its institutions were being organized?” We are inspired. What would medicine—its training, practice, and presumption—look like if it were informed by the scholarship and experiences of a vast diversity of people: people who are racially-marginalized, sociologists, community organizers, queer, differently-abled?

Though medical education has made strides to address health disparities, these efforts are falling short. The burdens of racism are indisputable. Physicians are taught to train their eyes on the numbers, but without critical frameworks, they will continue to perseverate on health inequality ineffectively. They will know *about* health disparities, but be unable to articulate health inequities well enough to challenge them (6).

Health equity cannot be achieved through technologic advancement or market-based ingenuity. It is, fundamentally, not a problem of science, but an issue of ethics and justice. Indeed, while 30,000 deaths could be prevented through medical innovation annually, eliminating excess mortality associated with education inequities would save 200,000 lives yearly (106). Remaining idle and ignorant renders our institutions complicit in an unjust system that makes our patients sicker. Medical trainees should receive robust, critical education that allows them to confront the forces that bolster health inequity. This requires the analytical and action-oriented pedagogical framework of Critical Race Theory.

Since its origins in jurisprudence, CRT has expanded into realms of education and public health (64, 66, 107–110). That CRT has been effectively incorporated into other domains to better address educational, health, and legal inequities demonstrates that incorporating CRT into medical pedagogy is necessary. Indeed, that CRT remains absent from physician education suggests that efforts to address racial inequity in medicine are lagging. It further underlines that MedCRT perspectives must be integrated in senior, administrative, and faculty-level continuing medical education—not just that of early trainees.

Medical education is a powerful site of action: Institutional commitment to equity can begin with improving how we teach and produce knowledge about inequity itself. The principles of Critical Race Theory are especially equipped to train learners to see spiders that weave political economy and power together to create injustice. We urge medical institutions and educators to mobilize greater engagement with Critical Race Theory and take a decisive step toward a more equitable future for students and patients alike.

## Author Contributions

JT was responsible for conceptualization, research, original writing, and revisions. EL and KB was responsible for research, original writing, and revisions. All authors contributed to the article and approved the submitted version.

## Conflict of Interest

The authors declare that the research was conducted in the absence of any commercial or financial relationships that could be construed as a potential conflict of interest.
